# Wnt signaling pathway activities may be altered in primary Sjogren’s syndrome

**DOI:** 10.3906/sag-2102-367

**Published:** 2021-08-30

**Authors:** Ahmet KARATAŞ, Zühal ÖMERCİKOĞLU, Burak ÖZ, Adile Ferda DAĞLI, Onur ÇATAK, Fazilet ERMAN, Kazım ŞAHİN, Nevzat GÖZEL, Süleyman Serdar KOCA

**Affiliations:** 1 Department of Rheumatology, Faculty of Medicine, Fırat University, Elazığ Turkey; 2 Department of Internal Medicine, Aybasti Hospital, Ordu Turkey; 3 Department of Pathology, Faculty of Medicine, Fırat University, Elazığ Turkey; 4 Department of Ophthalmology, Faculty of Medicine, Fırat University, Elazığ Turkey; 5 Department of Medical Biochemistry, School of Medicine, Fırat University, Elazığ Turkey; 6 Department of Animal Nutrition, Faculty of Veterinary Science, Fırat University, Elazığ Turkey; 7 Department of Internal Medicine, Faculty of Medicine, Fırat University, Elazığ Turkey

**Keywords:** Sjögren syndrome, wingless, sclerostin, dickkopf -1

## Abstract

**Background/aim:**

Sjögren’s syndrome (SS) is an autoimmune disease and its pathogenesis is still not completely clear. The wingless (Wnt)/β-catenin pathway has recently been shown to play an important role in inflammation. This study aims to determine the serum and saliva levels of Dickkopf (DKK)1 and sclerostin and to evaluate Wnt-1 and Wnt-3a expression in the salivary gland in patients with primary SS.

**Materials and methods:**

This study included 30 patients diagnosed with SS, 30 patients diagnosed with systemic lupus erythematosus (SLE), and 29 healthy controls. Serum and saliva levels of DKK1 and sclerostin were measured and the expressions of Wnt1 and Wnt3a in the salivary gland were measured immunohistochemically.

**Results:**

Serum DKK1 and sclerostin levels were lower in the SS and SLE groups compared to the control group (both p < 0.001). Saliva DKK1 levels were higher in the SS group compared to the control and SLE groups (p = 0.004 and p = 0.009, respectively). Wnt1 and Wnt3a expression were found in salivary gland tissue samples in 71.4% of primary SS patients and relatively frequent than control group.

**Conclusions:**

Serum DKK1 and sclerostin levels in primary SS and SLE were decreased. Moreover, levels of Wnt1 and Wnt3a expression in the salivary gland were also elevated in primary SS. Therefore, it can be concluded that the Wnt/β-catenin pathway activities may be altered in case of glandular inflammation.

## 1. Introduction

Sjögren’s syndrome (SS) is a systemic autoimmune disease that is prevalent in women [1,2]. Its pathogenesis is not completely clear; however, genetic disposition, viral stimulation, hormonal factors, and natural and acquired immunity have been implicated [36]. The chief symptoms of the disease include dryness in the mouth and eyes, fatigue, and joint pain due to exocrinopathy. These symptoms, particularly fatigue, decrease the patient’s life quality and result in an impairment of cognitive capacity [79]. Currently, there is no completely effective treatment for these symptoms [10]. Thus, determining the pathogenesis of this disease is important. Accordingly, studies are being conducted to identify the pathologic pathways.

The (Wnt) signaling pathway plays an important role in the development of the immune system, at the organogenesis stage, and in the regulation of various biological events such as the proliferation and differentiation of cells [11,12]. The Wnt/Wingless β-catenin signaling pathway is activated by proteins such as low-density lipoprotein receptor-related protein (LRP) 5, LRP6 and frizzled, and inhibited by extracellular proteins such as sclerostin and Dickkopf (DKK) 1 [1216]-. Studies have been conducted on the Wnt pathway in relation to various rheumatic diseases such as rheumatoid arthritis (RA), spondyloarthritis, and systemic lupus erythematosus (SLE); and this pathway was shown to be linked to the pathogenesis [17,18]. 

The present study aims to evaluate the serum and saliva levels of sclerostin and DKK1, and to immunohistochemically evaluate the levels of Wnt1 and Wnt3a expression in the salivary gland, in patients with primary SS. Moreover, the relationship between these parameters and clinical activity were also determined.

## 2. Materials and methods

### 2.1. Study subject

The study was carried out by the departments of rheumatology at FiratUniversity. Ethical committee approval was obtained from FiratUniversity clinical research ethics committee. We conducted our study in accordance with the approved guidelines of ethical principles for medical research involving human subjects. Written informed consent was provided from all participant. This study included 30 primary SS patients, 30 patients diagnosed with SLE (as a patient control group), and 29 healthy controls.

The SS and SLE patient groups were composed of patients who applied to the rheumatology outpatient clinic, met the respective diagnostic criteria [19,20], and agreed to participate in the study. The healthy control group was composed of volunteers who applied to the rheumatology outpatient clinic but did not have rheumatic inflammatory pathology according to the evaluations. Individuals younger than 18 years and older than 65 years; pregnant women; breastfeeding women; and patients with active infections, poorly controlled diabetes, heart failure, and malignant diseases were excluded from the study.

In addition, archived salivary gland tissue samples of 14 patients, diagnosed with SS and included in the study, and of 12 patients who had provided salivary gland tissue specimens for a preliminary diagnosis of SS but were not diagnosed with SS (pathological control group) were included in the study to be evaluated immunohistochemically for Wnt1 and Wnt3 expression levels. Therefore, time to harvest salivary gland tissue samples was different time periods from saliva and serum sample harvesting.

### 2.2. Clinical Evaluation

The patients were evaluated with regard to the parameters of disease activity used in the follow-up of SS and SLE. Disease activity was determined using ESSDAI (EULAR SS disease activity index) and ESSPRI (EULAR SS patient reported index) in SS patients [21], and using SLEDAI (systemic lupus erythematosus disease activity index) in SLE patients [22].

### 2.3. Laboratory evaluation

The results of the routine tests (fasting blood sugar, creatinine, ALT, blood count, erythrocyte sedimentation rate [ESR], and C-reactive protein [CRP]) were recorded in the patient and the control groups. Additionally, for the SS group, results indicating ANA, anti-Ro, anti-La; and for the SLE group ANA, anti-dsDNA, anti-Sm, C3 and spot urine protein were recorded from the patient files. In addition to these routine tests, 5blood and 2m-ml - saliva samples were collected from all participants after overnight fasting. Blood samples were centrifuged at 3000 rpm for 5 , and 2 m of serum was separated. The obtained serum and saliva samples were stored at 20 lminutesl-C until the day of the analysis. the end of the study, the stored samples were assessed to determine serum and saliva levels of sclerostin and DKK1 with the ELISA method, using an appropriate commercial kit °In (YH Biosearch, Pudong District, China).

### 2.4. Histopathological evaluation

Salivary gland tissue samples of 14 SS patients that had been collected for diagnostic purposes, and archived tissue samples of 12 patients who had provided minor salivary gland tissue samples due to a preliminary diagnosis of SS but were not diagnosed with SS (pathology control group) were included in the study. Tissue specimens were paraffin embedded. Formalin-fixed paraffin-embedded tissue sections that were cut 3-μm thick were placed onto positively charged glass slides (Isotherm, Objektträger, Braunschweig, Germany) .Immunohistochemical (IHC) staining was performed using tissue sections to determine Wnt1 and Wnt3a expression. The following antibodies were used for immunohistochemistry: Wnt1 (Mouse Wnt1 primary antibody (10C8), NBP1-51575, Novus biologicals, USA) and Wnt3a (Mouse Wnt3a primary antibody, NBP1-74183, Novus biologicals, USA). IHC staining of tissue sections were used the Ventana BenchMark Ultra Autostainer (Ventana, Tuscon, AZ-85755, USA) and the UltraView Univerversal DAB kit (Ventana, Tuscon, AZ-85755, USA), following the manufacturer’s instructions. Tissue sections were imaged with an Olympus B×51 upright light microscope (Olympus America, Center Valley, , USA) and images captured in digital color camera (DP71, Olympus) 

PennsylvaniaIn the evaluations, the criteria for positivity was defined as cytoplasmic staining in the ducts. The findings were graded based on staining intensity as follows; [23]

-: no staining 

+: mild staining 

++: moderate staining 

+++: strong staining2.5. **Statistical analysis**

The continuous data obtained in the study were presented as mean ± standard deviation, while nonparametric data without normal distribution were expressed as median (minimum-maximum). Statistical analyses were conducted using the IBM-SPSS, 22.0 software (International Business Machines-Statistical Product and Service Solutions (version )IBM Corp., Armonk, NY, USA). The Chi-square test was used for categorical data. Normal distributions were tested with the KolmogorovSmirnov test. The significant difference among groups was determined by the KruskalWallis variance analysis and the MannWhitney U test for dual comparisons in the nonnormal distributed and nonparametric data. One-way ANOVA and the student’s t-test for dual comparisons were performed parametric data with normal distribution. Bonferroni correction was applied since there were three study groups. Analysis of covariance (ANCOVA) was also used to adjust variables for age and . P-values < 0.05 were considered significant.

## 3. Results

### 3.1. Baseline characteristics

The demographic and clinical data of the study groups were presented in the Table 1. The median (min-max) age was determined as 51 (3665) -years for the SS group, as 37 (2250) -years for the SLE group, and 29 (2357) -years for the healthy control group. The percentage of females in the healthy control, SLE, and SS groups were determined as 51.7%, 80%, and 100%, respectively. ANCOVA was used to adjust variables for age and , since the differences between the groups in terms of age and gendergender were statistically significant (both p < 0.05). The median (min-max) disease duration was 1.7 (011) years for SS patients and 1.5 (013) years for the SLE group. The groups did not differ the disease durations (p > 0.05). 

**Table 1:** Demographic and clinical data of the study groups.

Data×	HC(n = 29)	SLE(n = 30)	SS(n = 30)	P1*	P2*	P3*
Age, years	29 (23–57)	37 (22–50)	51 (36–65)	0.068	<0.001	0.001
Disease duration, years	-	1.7 (0–11)	1.5 (0–13)	-	-	0.738
Female, %	51.7	80	100	0.029	< 0.001	0.023
Hemoglobin, g/dL	14.7 ± 1.2	12.4 ± 2.1	13.3 ± 1.5	< 0.001	0.004	0.104
WBC, 103/µL	7.2 ± 1.9	6.2 ± 2.4	5.9 ± 1.8	0.140	0.069	0.907
ESR, mm/h	5 (1–39)	18.5 (3–66)	12 (5–71)	<0.0001	<0.0001	0.361
CRP, mg/dL	3.4 (3–16.8)	3.4 (1.9–14.6)	3.4 (3.2–70)	0.612	0.368	0.778
Serum DKK1, ng/mL	52.9 (24.1–74.5)	26.5 (2.1–63.2)	33.9 (20.6–59)	< 0.001	< 0.001	0.046
Saliva DKK1, ng/mL	30.6 ± 5.9	31.1 ± 6.9	36.3 ± 6.9	0.944	0.004	0.009
Serum sclerostin, ng/mL	15.5 (3.4–18.3)	4.6 (2.1–18.1)	4.5 (2–18.6)	< 0.001	< 0.001	0.984
Saliva sclerostin, ng/mL	16.1 ± 3.4	15.7 ± 2.5	15.6 ± 3.7	0.877	0.838	0.996

### 3.2. Basic laboratory tests

Hemoglobin levels were significantly lower in the SS and SLE groups compared to the healthy control group (p = 0.004 and p < 0.001, respectively). However, there were no statistically significant differences with regard to leukocyte, platelet, and CRP levels. ESR was higher in the SLE and SS groups compared to the control group (p < 0.001 for both).

### 3.3. Disease activity score of SLE and SS patients

Systemic lupus erythematosus patients showed a median SLEDAI score of 10 (029), -and SS patients showed a median ESSDAI score of 1 (03)-. ANA positivity was 96% in the SLE group and 83.3% in the SS group.

### 3.4. The sclerostin and DKK1 levels of the patient and healthy control groups

The SS group demonstrated lower serum DKK1 and sclerostin levels compared to the control group (both p < 0.001), even after adjustment for age and by ANCOVA analysis. However, saliva levels of DKK1 and sclerostin were comparable across the SS and healthy control groups (p > 0.05). Serum DKK1 and sclerostin levels were lower in SLE patients compared to healthy controls (p < 0.001 for both). However, the SLE group did not differ from the control group in terms of saliva DKK1 and sclerostin levels. In addition, serum and saliva levels of DKK1 were higher in patients diagnosed with SS compared to the SLE group (p = 0.046 and p = 0.009, respectively). However, the SS and SLE groups were not significantly different with regard to serum and saliva levels of sclerostin. 

A positive correlation was determined between serum DKK1 levels and serum sclerostin levels in the healthy control, SLE, and SS groups (respectively; r = 0.829, p < 0.001; r = 0.783, p < 0.001; and r = 0.677, p < 0.001). Serum and saliva levels of sclerostin and DKK1 were not correlated with ESSDAI and SLEDAI in these groups (p > 0.05).

### 3.5. Wnt1 and Wnt3a expression were increased in the salivary gland of the patients with SS

**Figure 1:**
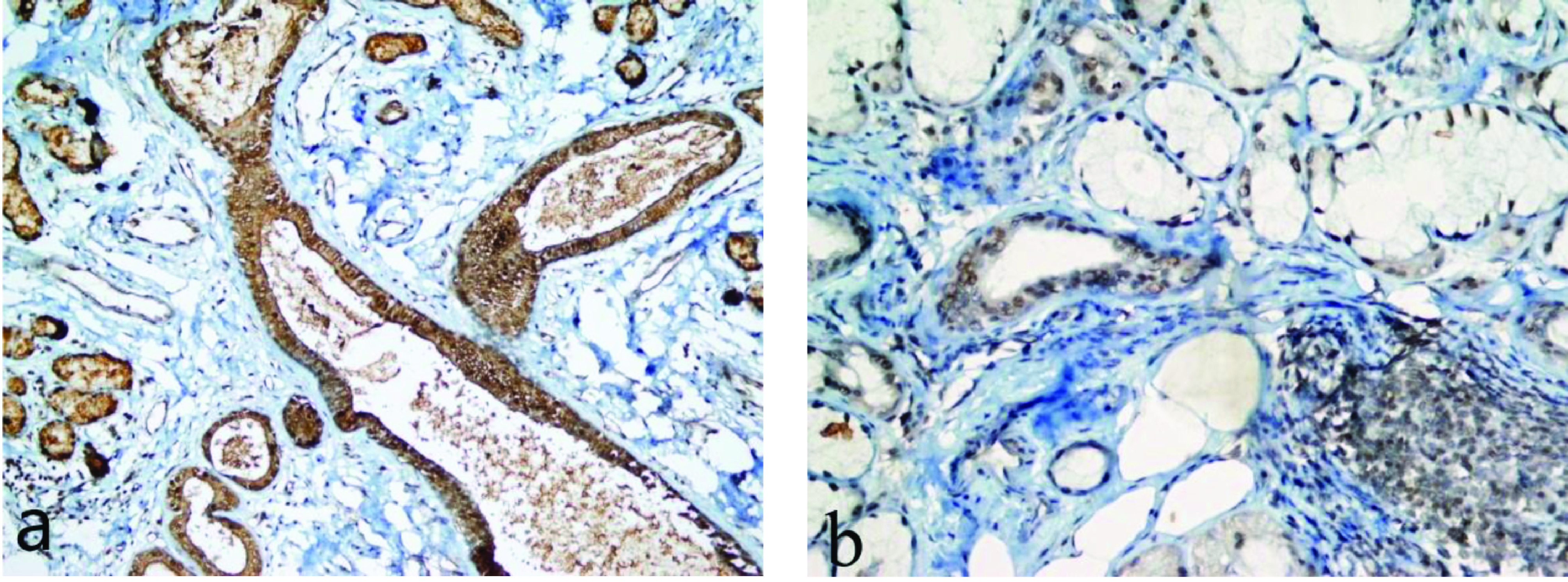
Immunohistochemical Wnt1 expression in the salivary gland. (a) Strong nuclear and cytoplasmic Wnt1 positivity in the ducts and acini in controls (×400), (b) Mild nuclear and cytoplasmic Wnt1 positivity in the ducts and acini in SS patients (×400).

**Figure 2:**
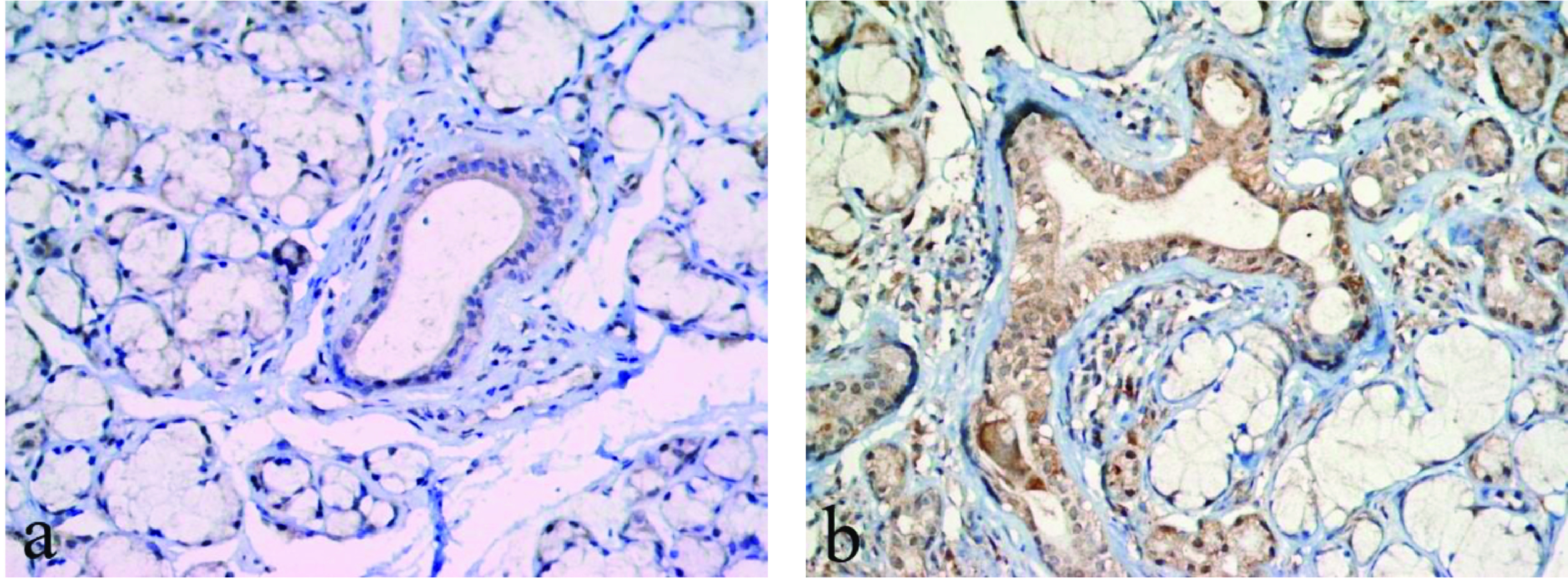
Immunohistochemical Wnt3a expression in the salivary gland. (a) Mild nuclear and cytoplasmic Wnt3a positivity in the ducts and acini in controls (×400), (b) Strong nuclear and cytoplasmic Wnt3a positivity in the ducts and acini in SS patients (×400).

Wnt1 and Wnt3a positivity were higher in the salivary gland biopsy samples of patients diagnosed with SS compared to the control group (Figures 1a, 1b for Wnt1; Figures 2a, 2b for Wnt3a). However, this difference was not statistically significant (Table 2). 

**Table 2:** Wnt1 and Wnt3a expressions in the salivary glands of patients with primary SS.

	Control	Sjögren’s Syndrome	P*
Wnt1 positive patients, %	46.2	71.4	0.182
Mild, %	7.7	42.9	
Moderate, %	15.4	14.3	
Strong, %	23.1	14.3	
Wnt3a positive patients, %	53.8	71.4	0.345
Mild, %	30.8	7.1	
Moderate, %	15.4	14.3	
Strong, %	7.7	50	

SS patients with Wnt1- and Wnt3a-positive salivary glands and those with Wnt1- and Wnt3a-negative salivary glands did not have significantly different serum and saliva levels of DKK1 and sclerostin (for each p > 0.05). Similarly, Wnt1- and Wnt3a-positive SS patients and Wnt1- and Wnt3a-negative SS patients were not significantly different with regard to ANA, anti-Ro, and anti-La antibody positivity (for each p > 0.05) (Table 3).

**Table 3:** Differences between Wnt1- and Wnt3a-positive and Wnt1- and Wnt3a-negative patients with primary SS.

	Wnt1			Wnt3a		
Data×	Negative	Positive	P*	Negative	Positive	P*
Anti-Ro titre, IU/L	131.2 ± 79.9	80.4 ± 95	0.1	98.2 ± 96.6	93.6 ± 2	0.7
Anti-La titre, IU/L	6.5 ± 2	76.7 ± 107.8	0.7	112.2 ± 134.3	32.6 ± 68.8	0.3
Serum DKK1, ng/mL	28.6 ± 5.8	36.9 ± 9	0.06	31.9 ± 4.3	35.6 ± 10.2	0.6
Saliva DKK1, ng/mL	38 ± 10.5	35.8 ± 7.2	0.8	37.9 ± 9.5	35.8 ± 7.7	0.6
Serum sclerostin, ng/mL	4.5 ± 2.5	5.2 ± 4.8	1	3.8 ± 1.1	5.5 ± 4.9	0.6
Saliva sclerostin, ng/mL	14.7 ± 2	14.2 ± 4.9	0.6	15.5 ± 1.7	13.9 ± 4.8	0.3

## 4. Discussion

The aim of this study was to determine the activity of Wnt/β-catenin signaling pathway in primary SS. For this purpose, serum and salivary sclerostin and DKK1 levels and Wnt1 and Wnt3a immunohistochemical expressions in salivary gland tissue were evaluated.

SS is a chronic, autoimmune disease that mainly affects the tear and salivary glands. Lymphocyte infiltration and dysfunction of the exocrine glands result in dry mouth and eyes [24]. Hereditary and environmental factors have been implicated in the pathogenesis. One of the most important theories in the pathogenesis is that the SS is a process that starts with epithelitis and then continues with lymphocyte infiltration. Therefore, the disease is considered by some researchers as autoimmune epithelitis [25]. Moreover, recent data show that salivary gland epithelial cells play an active role in initiating inflammatory and autoimmune response [26].

Wnt/β - catenin signaling pathway plays an important role in embryonic development, tissue homeostasis, cellular proliferation and also the regulation of the immune system such as T, B and dendritic cells [27,28]. Genes associated with this pathway have been expressed in a wide variety of cell types and tissues such as adipocytes, osteoblasts, platelets, lymph gland, adrenal gland, thyroid, pituitary and salivary gland [29,30]. Wnt/β-catenin signaling pathway is known to play a role in the pathogenesis of cancer and autoimmune diseases [28,3134]. In this respect, there are a number of studies investigating biomarker in these diseases [35,36]. Wnt/-β-catenin signaling pathway is an important regulatory pathway in bone formation and destruction [37,38]. 

The blocking DKK1, which is an inhibitor of Wnt/β-catenin signaling pathway, decreased osteoclastic activity and decreased bone erosion in RA patients [39]. It has also been found that DKK1 levels increased in RA patients and correlated with bone erosion [37]. Wehmeyer et al. [40] have found that the sclerostin levels in the synovium of RA are significantly higher than in patients with osteoarthritis. In a subsequent study [41], it has been observed that the level of DKK1 is higher in patients with RA and glucocorticoid treatment decreases serum DKK1 level.

In our study, it is observed that serum DKK1 and sclerostin levels are decreased in patients with primary SS. There are controversial results in terms of DKK1 and sclerostin levels in inflammatory diseases. Contrary to the articles documenting increased serum DKK1 and sclerostin levels in RA [40,41], Zhang et al. [42] have reported that serum DKK1 level in patients with RA is similar in healthy subjects. There are two controversial study reporting increased [42] and decreased [43] serum DKK1 levels in patients with ankylosing spondylitis. 

Dovjak et al. [44] have found that DKK1 levels are correlated with age and the presence of osteoporosis. Furthermore, serum DKK1 level is higher in males. Gifre et al. [41] have documented that treatment alters serum DKK1 levels. Therefore, it can be speculated that the differences on demographical data, clinical manifestations and treatment modalities of the mentioned studies are the potential reasons of controversial results of DKK1 in inflammatory diseases. 

In our study, it is documented that serum the level of DKK1, an inhibitor of Wnt signaling pathway is decreased, while glandular tissue Wnt1 and Wnt3a expressions are relatively higher, in patients with primary SS. Wnt signaling pathways are an important regulator in salivary gland organogenesis. Wnt/β-catenin signaling plays a role in both mesenchymal and ductal maturation of salivary glands. On the other hand, the Wnt/β-catenin signal and the noncanonical Wnt pathway work together to regulate maturation in the morphogenesis stage [45,46]. However, the Wn/-t β-catenin signal is significantly activated in the channel epithelium during functional regeneration in the adult salivary gland [46]. Fernandez-Torres et al. [47] have found that gene polymorphisms such as LRP5, FRZB, and ADIPOQ associated with Wnt/β-catenin signaling pathway lead to increased risk of primary SS.

The present study has few limitations. The sample size was not calculated before enrolling study participants. Age and were not matched; however, adjustment for age and gender by ANCOVA was performed during statistical analysis. Moreover, salivary gland tissue and serum samples were not harvested same time.

In present study, Wnt1 and Wnt3a expression are found in salivary gland tissue samples in 71.4% of primary SS patients. High salivary DKK1 levels in primary SS group can be explained by glandular inflammation and damage. According to the best of our knowledge, the present study is the first to evaluate the efficacy of Wnt/gender β-catenin pathway in primary SS. In conclusion, in present study, serum levels of DKK1 and sclerostin, Wnt/β-catenin signaling pathway inhibitors, are decreased and Wnt1 and Wnt3a are accumulated in the glandular tissue in primary SS patients. These results indicate that Wnt/β-catenin signaling pathway is affected in primary SS.
